# Heat-induced female biased sex ratio during development is not mitigated after prolonged thermal selection

**DOI:** 10.1186/s12862-023-02172-4

**Published:** 2023-11-02

**Authors:** Marta A. Santos, Marta A. Antunes, Afonso Grandela, Ana Carromeu-Santos, Ana S. Quina, Mauro Santos, Margarida Matos, Pedro Simões

**Affiliations:** 1 cE3c – Centre for Ecology, Evolution and Environmental Changes & CHANGE – Global Change and Sustainability Institute, Lisboa, Portugal; 2 Departamento de Biologia Animal, Faculdade de Ciências, Universidade de Lisboa, Lisboa, Portugal; 3 CESAM – Centre for Environmental and Marine Studies, Universidade de Aveiro and Faculdade de Ciências, Universidade de Lisboa, Lisboa, Portugal; 4 Departament de Genètica i de Microbiologia, Grup de Genòmica, Bioinformàtica i Biologia Evolutiva (GBBE), Universitat Autònoma de Barcelona, Bellaterra, Spain

**Keywords:** Drosophila, Experimental evolution, Global warming, Climate change, Offspring sex ratio, Thermal adaptation, Thermal selection, Heat stress

## Abstract

**Background:**

The negative impacts of climate change on biodiversity are consistently increasing. Developmental stages are particularly sensitive in many ectotherms. Moreover, sex-specific differences in how organisms cope with thermal stress can produce biased sex ratios upon emergence, with potentially major impacts on population persistence. This is an issue that needs investigation, particularly testing whether thermal selection can alleviate sex ratio distortions in the long-term is a critical but neglected issue. Here, we report an experiment analyzing the sex ratio patterns at different developmental temperatures in *Drosophila subobscura* populations subjected to long-term experimental evolution (~ 30 generations) under a warming environment.

**Results:**

We show that exposure to high developmental temperatures consistently promotes sex ratio imbalance upon emergence, with a higher number of female than male offspring. Furthermore, we found that thermal selection resulting from evolution in a warming environment did not alleviate such sex ratio distortions generated by heat stress.

**Conclusions:**

We demonstrate that heat stress during development can lead to clear sex ratio deviations upon emergence likely because of differential survival between sexes. In face of these findings, it is likely that sex ratio deviations of this sort occur in natural populations when facing environmental perturbation. The inability of many insects to avoid thermal shifts during their (more) sessile developmental stages makes this finding particularly troublesome for population subsistence in face of climate warming events.

**Supplementary Information:**

The online version contains supplementary material available at 10.1186/s12862-023-02172-4.

## Background

The negative impacts of climate change on biodiversity are consistently increasing. Skewed sex ratios generated by sex-specific differences in how organisms tolerate thermal stress is one of such detrimental aspects, with potentially huge impacts on population growth, adaptive potential and ultimately species persistence [1, 2]. Recent studies have suggested that females are generally more heat tolerant than males [3, 4], but this is not always the case (e.g. see also [2, 5]). Studies in ectotherms, and insects in particular, have clearly shown that there is ontogenetic variation in thermal sensitivity, with developmental stages generally being more vulnerable to thermal stress (e.g. [2, 6–13]). Specifically, studies in holometabolous insects revealed consistent higher thermal resistance in the pupal than egg and larval stages (e.g. [14–16]). An additional and related problem is the possible sex ratio distortion caused by increasing temperatures during development [2], which can impact on effective population size and, hence, on adaptive potential because theory predicts a close association between effective population size and additive genetic variance ([17]; but see [18]). However, most research on the effect of temperature on sex ratio has been conducted in ectotherms in which sex determination during development is temperature-dependent [2]. Further investigation is needed in order to understand whether sex differences in thermal tolerance and mortality occur at those early stages and how these affect sex ratio. If sex-biased mortality occurs during development, the resulting skewed offspring sex ratio can cause major detrimental impacts on the persistence of populations. Such impact will likely be more harmful with increasing distortions in sex ratio [19], which in turn will depend on the severity and duration of the thermal stress the populations suffer. Theoretical models suggest that sex ratio variation can have important impact on population growth and extinction risk, being dependent on factors such as the population mating system [20, 21]. Specifically, Lee et al*.* [21] show that the optimal sex ratio for population growth can vary substantially between monandrous (~ 0.5) and polygynous populations (~ 0.85, female-biased). In the case of monandrous populations, sex ratio distortions in the order of 0.1 can expectedly lead to around 30% decline in population growth.

Evidence for the impact of developmental temperature on sex ratio in insects is not straightforward. In *Drosophila melanogaster*, Kristensen et al. [22] found a female-biased sex ratio in emerging flies following development at low temperatures, possibly due to higher juvenile mortality of males (see also [23], for similar finding in Hemiptera). Evidence for male-biased sex ratio upon emergence at higher temperatures has been reported in the harlequin ladybird, *Harmonia axyridis* [24]. However, a recent study using lines from the Drosophila Genetic Reference Panel (DGRP) did not find an effect of benign or heat stress fluctuating developmental temperatures on sex ratio [25]. Biased sex ratios were also not found at several developmental stages upon exposure to several temperatures in *D. melanogaster* populations [26]. These mixed results underscore the need of gathering additional data to enable more accurate predictions regarding sex ratio distortion upon developmental thermal stress as a result of climate change.

Assuming that rising temperatures might disrupt a population sex ratio in holometabolous insects, a relevant question that deserves investigation is to what extent thermal selection can alleviate such sex ratio distortions in the long-term [2]. Using the power of Experimental evolution [27, 28], several studies have addressed the response of populations under increasingly warmer conditions (e.g. [29–31]). However, to our knowledge none have tackled the impact of heat stress on sex ratio bias and the effect of thermal selection to putative alleviate such effect.

The largely monandrous species *Drosophila subobscura* [32] is a classic case study of thermal adaptation in ectotherms, with ample geographical variation for inversion polymorphisms that shifted globally as a result of climate warming, providing compelling evidence for their adaptive role [33–35]. Thermal plasticity has been reported for several relevant traits such as reproductive performance [10, 13, 36–38] and thermal tolerance [39, 40]. Evidence for evolutionary responses to varying thermal conditions have also been described in this species for thermal tolerance [41], locomotor behavior [42] and reproductive performance ([43], see below).

Our team has been addressing the evolutionary changes in reproductive performance of *Drosophila subobscura* populations that are evolving in a warming thermal selection regime [38, 43] following lab adaptation (i.e. evolutionary experimental domestication, [44]). We have analyzed two sources of historically differentiated populations, one from higher latitudes (Northern Europe) and another from lower latitudes (Southern Europe) – [45]. We found evolutionary changes in reaction norms as a result of thermal selection, with populations from higher latitude evolving under warming conditions showing better reproductive success than controls (kept at the ancestral temperature) at stressful high temperatures [43]. As such, here we focus on this population to address the effect of such stressful conditions during development on offspring sex ratio upon emergence. We will specifically test whether thermal selection can mitigate potential sex ratio bias by improving juvenile survival of the least tolerant sex. If development under thermal stress causes a change in the expected 1:1 offspring sex ratio, we expect this deviation to be lower in the warming regime populations compared to their respective controls provided thermal selection has acted to reduce excess mortality in the development stages of the more sensitive sex.

## Results

**Fig. 1:**
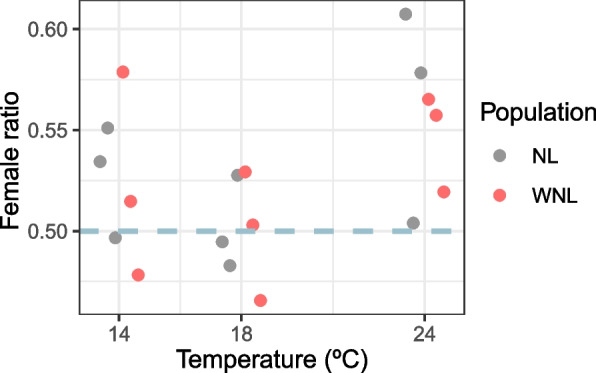
Female ratio upon emergence at lower, intermediate and higher temperatures for warming and control populations (WNL and NL respectively). Legend: Data points represent the mean female ratio for each of the three replicate populations (WNL_1-3_ and NL_1-3_). Female ratio was calculated as the ratio between female offspring number and total offspring number

We first tested for the overall effects of Sex, Selection and Temperature on offspring number. We found significant differences in the number of male and female offspring (factor *Sex*, see Table 1), indicating a general bias in the offspring sex ratio relative to the 1:1 expectation (see also Fig. 1). Offspring sex ratio did not differ significantly between temperatures or selection regimes (*Sex x Temp* interaction and *Sex x Selection* interaction respectively, see Table 1), although a marginally non-significant effect was found for the *Sex x Temp* interaction (see Table 1). While no significant overall effects of thermal selection were found (factor *Selection*, see Table 1), we observed that differences in total offspring number between thermal selection regimes varied significantly across temperatures (significant *Selection x Temp* interaction, see Table 1). This corresponded to a higher offspring number (reproductive success) in the warming populations relative to their controls at 24 ºC but not at the other temperatures (see Table S1), a finding that was already reported in our previous paper focusing on the reproductive success of these populations (see Santos et al. [43] and introduction).

**Table 1:** Analysis of the effect of sex, selection and temperature on offspring number

Model parameters	d.f	*χ*2
Sex	1	7.837**
Temp	2	45.738***
Selection	1	0.545 n.s
Sex x Temp	2	4.633 m.s
Sex x Selection	1	0.175 n.s
Selection x Temp	2	22.915***
Sex x Temp x Selection	2	0.109 n.s

Significance levels: *p* > 0.1 n.s.; 0.1 > *p* > 0.05 m.s.; 0.05 > *p* > 0.01*; 0.01 > *p* > 0.001**; *p* < 0.001***

Considering the general sex ratio bias, and the suggestion that this deviation could vary between temperatures (overall model and data for each replicate population, see Table S1 and Fig. 1), we assessed sex ratio differences at specific temperatures. First, we tested the general expectation of no sex ratio bias at benign, control (18 ºC) conditions. We confirmed that at 18 ºC the sex ratio did not deviate from the 1:1 expectation (factor *Sex*). Also, there was no significant effect of selection or its interaction with sex (Table S2).

We then tested for significant deviations from a 1:1 sex ratio at extreme temperatures (see Table 2). At 24 ºC, we found a significant excess of female offspring (Factor *Sex*, see Table 2). A significant effect of selection was also found (Factor *Selection*, Table 2), with a higher number of offspring in the warming populations relative to their controls (see Table S1). On the other hand, at 14 ºC there was no significant deviations to the 1:1 sex ratio (Factor *Sex* was not significant, see Table 2). A significant effect of selection was found at this temperature (Factor *Selection*, Table 2), with a lower number of offspring in the warming populations relative to their controls (see Table S1).

**Table 2:** Analysis of the effect of sex and selection on offspring number at extreme temperatures (14 ºC and 24 ºC)

Model parameters	d.f	*χ*2—14 °C	*χ*2—24 °C
Sex	1	2.722 n.s	5.610*
Selection	1	4.230*	14.712***
Sex x Selection	2	0.062 n.s	0.132 n.s

Significance levels: *p* > 0.1 n.s.; 0.1 > *p* > 0.05 m.s.; 0.05 > *p* > 0.01*; 0.01 > *p* > 0.001**; *p* < 0.001***

## Discussion

### High developmental temperatures can lead to female-biased sex ratios

In this study we show that heat stress during developmental temperatures can induce shifts in the offspring sex ratio upon emergence. A female bias was observed when development occurred at a temperature 6 ºC higher than control. This assay temperature of 24 ºC is well within the range of developmental temperatures that *Drosophila subobscura* can withstand, being around 3 ºC below the upper limit for juvenile viability [39, 46]. In any case, the intensity of the heat stress suffered by our populations was high, with controls and warming populations showing a drop in reproductive success of around 64% and 32% relative to benign conditions, respectively [43].

Sex-biased thermal tolerance is one mechanism by which temperature can impact on sex ratio, with other more thoroughly studied mechanisms being temperature-dependent sex determination, and temperature-induced sex reversal [2]. The female-biased sex ratio we report here is likely due to a lower heat tolerance of males during development, causing higher male mortality. This is in line with the general expectation of a higher heat tolerance of females than males [2, 3]. Increased larval density can potentially lead to female skewed sex ratios [47], although no consistent trend on the effect of density on animal sex ratio has been found [48]. However, in our study male-biased mortality (and mortality in general) due to differences in juvenile density between temperatures could be ruled out because egg densities in the assays were about the same as those applied during the regular maintenance of populations. There are some mixed results in the literature concerning the impact of temperature on offspring sex ratio upon emergence in insects. Evidence for a female bias at lower temperatures was reported in *Drosophila melanogaster* [22], due to differential juvenile mortality between sexes. Nevertheless, other studies showed an absence of deviations from the expected 1:1 sex ratio also in *D. melanogaster* [25, 26], and still others a male bias at higher temperatures (in *Harmonia axyridis*, [24]). Altogether, these results suggest variation in sex-specific developmental thermal tolerance between species or even populations that might involve different factors such as body size for instance [2]. In addition, differences in the specific thermal treatment applied between studies (e.g. intensity and duration of thermal stress) may also be a relevant factor in explaining the observed differences.

While there is evidence in insects that some stages of the developmental process are more sensitive to heat stress than others (e.g. [7–9]), measurements of sex differentiation in heat tolerance are conspicuously missing in discriminating stages [2]. In our study we also could not pinpoint which specific developmental stage was particularly responsible for the sex-bias we observed, though *Drosophila* larvae have been shown to be more sensitive to heat stress than pupae [14, 16].

The observed sex ratio bias in our study – an average of 55.5% emerging females at higher temperature – is comparable to that found by Walsh et al. [49] in *Drosophila virilis*. In that study, an operational sex ratio bias of 44% of fertile males—relative to the total number of fertile adults—was found, following a sub-lethal heat shock of 38 °C for four hours during the pupal stage. These results highlight the negative impact of heat stress on male thermal tolerance and fertility – see below. 

### Sex ratio distortion does not respond to thermal selection

A lower sex ratio distortion in warming populations relative to controls was expectable at higher temperatures if the higher reproductive success of the former populations (also relative to controls; reported in [43]) was associated with a lower juvenile male mortality than that in the controls. However, the bias towards females at high temperature was similar in both warming and control populations, suggesting no improvement in male mortality (relative to female mortality) under stress in warming populations. Thus, our study does not support the possibility that populations respond to thermal selection by reducing sex ratio bias under thermal stress. Further studies should also address whether thermal selection could mitigate sex ratio distortions generated under more ecologically meaningful environmental scenarios such as heat waves and thermally variable environments rather than constant temperatures.

It is important to notice that the distortions in sex ratio we report here occurred immediately following emergence, thus reflecting variation in thermal tolerance during developmental stages and not sex-biased adult mortality by heat stress. To our knowledge, the direct impact of sex ratio deviations due to sex-biased heat mortality on population persistence has not been assessed empirically. Results from a theoretical study by Lee et al*.* [21] suggest that a skew of around 10% in sex ratio of a monandrous population (towards either male of female bias) could have a substantial negative impact on population growth (~ 30% decline). If that is the case, the impact of sex ratio deviations of the magnitude we report here can have negative consequences on natural populations.

Sex ratios during adult stage might be further skewed towards females following heat wave events, given the increasing evidence for sex differences in thermal fertility [3]. In fact, imbalance in sex ratio during the adult stage has been shown to occur through cryptic male sterility promoted by heat stress, with a lower number of fertile males relative to females (see [49] and above). The possibility to mitigate the impact of such sex ratio deviations in nature will depend on the ability of males to recover their fertility in a reasonable time window considering the species life cycle and also on the ability of females to find and (re)mate with less affected males.

## Conclusions

Our present findings argue that heat stress during development can be a driver of sex ratio imbalance at the start of a new generation. This represents an additional source of disturbance to populations sexual selection and fertility in the context of climate change [50]. Importantly, we did not find that populations evolving under heat stress can alleviate that bias after prolonged thermal selection. The inability of many insect species to avoid sudden thermal shifts during long, sessile developmental stages due to fewer opportunities for behavioral thermoregulation makes this finding particularly troublesome for the efficacy of reproduction in natural populations under increasing temperatures.

## Methods

### Population maintenance and thermal selection regimes

The initial *Drosophila subobscura* populations of this study derived from collections in 2013 in Groningen, The Netherlands (53º13′ N). The laboratory populations were designated NL and were three-fold replicated in the lab generating the NL_1-3_ populations. These were maintained in discrete generations with a 28-day cycle, 12L:12D photoperiod, at 18 ºC with controlled densities in both adult (~ 40 flies per vial) and juvenile stage (~ 70 eggs per vial)—the control conditions (see also [45]).

After 70 generations of lab evolution, a global warming regime started (Warming populations, WNL_1-3_) – see [38]. This thermal selection regime includes a daily fluctuation initially between 13 ºC and 21 ºC with an increase of 0.18 ºC per generation in daily mean and 0.54 ºC in daily amplitude. The NL populations are the controls as they represent the ancestral state for the new thermal regime.

Except for the thermal cycles defined above, all experimental populations were subjected to the same environmental conditions and manipulation. Census sizes were generally around 1000 for both selection regimes with some exceptions, the most important of which being the clear drops in population size by generations 22 and 24 (with 130 individuals being the lowest census size in a given replicate population) due to high mortality in the juvenile stages. Because of this, the temporal increases in thermal mean and amplitude in the warming cycle had to be stopped by generation 22. Since then, the warming populations have been kept in the same thermal cycle every generation with a mean temperature of 21.4 ºC, and lower and upper thermal extremes of 13.5 ºC and 29.4 ºC, respectively.

### Thermal plasticity assay

The aim of this study was to analyze the impact of different developmental temperatures on offspring sex ratio. The new data for this study—offspring sex ratio data—was obtained from a thermal plasticity assay performed after 31 generations of thermal selection [43] – see details below. In that study we addressed fecundity and productivity but did not discriminate offspring sex, the focus of the present study.

The assay involved the three replicate populations for each thermal selection regime (warming vs. control). It was performed in a block design, with each block corresponding to the set of same-numbered replicate populations that were simultaneously distributed and manipulated in the same experimental racks, e.g. Block 1 included samples from NL_1_, and WNL_1_ populations. All populations were maintained for one full generation in a common-garden environment, under control conditions (18ºC and 28-day life cycle) prior to the assay to reduce maternal effects.

Three lifelong temperature treatments were assayed: colder (14 ºC), intermediate (18 ºC), and warmer (24 ºC) temperature. Sixteen pairs of recently emerged virgin males and females were formed per population and temperature treatment and maintained as adults for 8 days, allowing to measure fecundity. Eggs laid during a 24-h egg laying period from all assayed couples at the eighth day of assay (8-day old flies) were allowed to develop under the same environmental conditions as experienced by assayed adults. Fecundity at day eight was consistently below 70 at all temperatures—the typical density used during the juvenile stages in the maintenance regime of our populations—so excess in egg density during development is not expected to impact on sex ratio estimates (as such density conditions will not lead to relevant juvenile mortality). As vials with very low total offspring number were not considered in the analysis (see below) this also excludes vials with extremely low egg density (below 5) from the analysis due to a high correlation between traits. As such, bias in our sex ratio estimates due to very low egg density is likely reduced in our analysis. The total number of offspring (imagoes) obtained for each couple after 10 days of screening since first emergence estimated the reproductive success. While the emergence of flies was not synchronous between treatments due to the effect of temperature on developmental time, a similar duration in the screening period was applied to all temperature treatments (10 days after first emergences in each treatment). Importantly, after day 8 of screening virtually no vial yielded new individuals in any of the treatments, so there was no data truncation due to extended developmental time. To estimate the offspring sex ratio for the present study, the total number of male and female offspring per vial was counted for each population – see Figure S1 for a schematic representation of the assay. A total of 6,103 individuals were screened to produce the new sex ratio data presented in this study. For more reliable estimates and to avoid distortions due to low sample size, only couples that had more than five offspring were considered in the analysis – see sample sizes (number of vials) per replicate population in Table S1 and the complete dataset in Table S3.

Populations from Southern Europe (PT_1-3_ and WPT_1-3_ – see [26, 31]), also assayed in the experiment, were excluded from the present study considering that (1) our aim is to test the possible evolution of offspring sex ratio concomitant with evolutionary improvement of reproductive success, which was not observed in these populations [43], and (2) there was a reduced sample size at higher temperatures in these populations, in particular WPT_1_ (with only one couple producing offspring).

### Statistical methods

Raw data for the analyses were the number of male and female offspring for each of the sixteen couples analyzed per replicate population and temperature treatment. Data was analyzed by applying a generalized mixed-effects model (GLMM) assuming a negative binomial distribution (Poisson and Quasi-Poisson distributions were also tested but not chosen due to higher Akaike Information Criterion (AIC) values in general). Type III Wald chi-square tests were used to obtain significance levels for differences between sexes, thermal selection regimes and temperature treatments, as well as their interactions.

The following overall model was applied (interactions with the random factor Block, also included in the model, are not presented for simplicity):

$$Y = \mu + Sex + Selection + Temp + Block + Sex \times Temp + Selection \times Temp + Sex \times Selection + Sex \times Selection \times Temp + \varepsilon$$

with *Y* being the number of offspring (males or females); *Sex,* the fixed factor for offspring sex (categories Female and Male); *Selection*, the fixed factor for thermal selection regimes (categories Control and Warming); and *Temp*, the fixed factor for the temperature treatments (14 ºC, 18 ºC and 24 ºC). *Block* was defined as the random effect, corresponding to the sets of same-numbered replicate populations from both thermal regimes. In this model, the factor *Sex* allows to directly test for deviations to the null expectation of equal number of male and female offspring (i.e. deviations in sex ratio). Models including either fecundity or productivity as covariates were applied to account for possible effects of variation in egg density and in reproductive success respectively on the sex ratio estimates but neither of these covariates were statistically significant and also had no effect on the statistical significance of the factors (and interactions) under analysis, so they were removed from the final models.

GLMM models with a negative binomial distribution were also specifically applied to 18 ºC data to test our a priori expectation of a 1:1 sex ratio in control conditions, and to test for deviation in sex ratio at the extreme temperatures. These models included *Sex* and *Selection* as factors and *Block* as random effect.

All statistical analyses were performed in R v4.0.4 using the *glmmTMB* package [51] for the generalized mixed linear models and *ggplot2* [52] package for data plots.

### Supplementary Information

**Additional file 1: Table S1.** Offspring sex ratio for each population and temperature. Offspring sex ratio was calculated as the female ratio, i.e. the female offspring number divided by the total offspring number. Total sample size (N) corresponds to the number of vials analysed for each replicate population.

**Additional file 2: Table S2.** Effect of Sex and Selection on offspring number at control (18 ^o^C) conditions.

**Additional file 3: Figure S1.** Schematic representation of the protocol used to assess the sex ratio after exposure to different thermal treatments. Legend: After 31 generations of thermal evolution individuals from both Control and Warming regimes were subjected to a one full generation common garden (18 ºC). After the common garden both selection regimes were submitted to one of three thermal treatments (14 °C, 18 °C or 24 °C). The emerging adults, formed in pairs, were assayed for fecundity at those same temperatures and the reproductive success was measured after a 10-day emergence period. In this study, a total of 6103 individuals were screened to assess the populations’ sex ratio.

**Additional file 4:  Table S3.** Raw data. Dataset with individual data - female and male offspring number for each temperature (14, 18 and 24 ºC), selection regime (WNL - warming vs NL - control) and replicate population (WNL1-3 and NL1-3).

## Data Availability

All data generated or analysed during this study are included in this published article [and its supplementary information files].
