# Supplementary figures and images for: Heat-induced female biased sex ratio during development is not mitigated after prolonged thermal selection
Source: BMC Ecol Evol. 2023 Nov 2;23:64. doi: 10.1186/s12862-023-02172-4 (PMC10623787; doi:10.1186/s12862-023-02172-4)

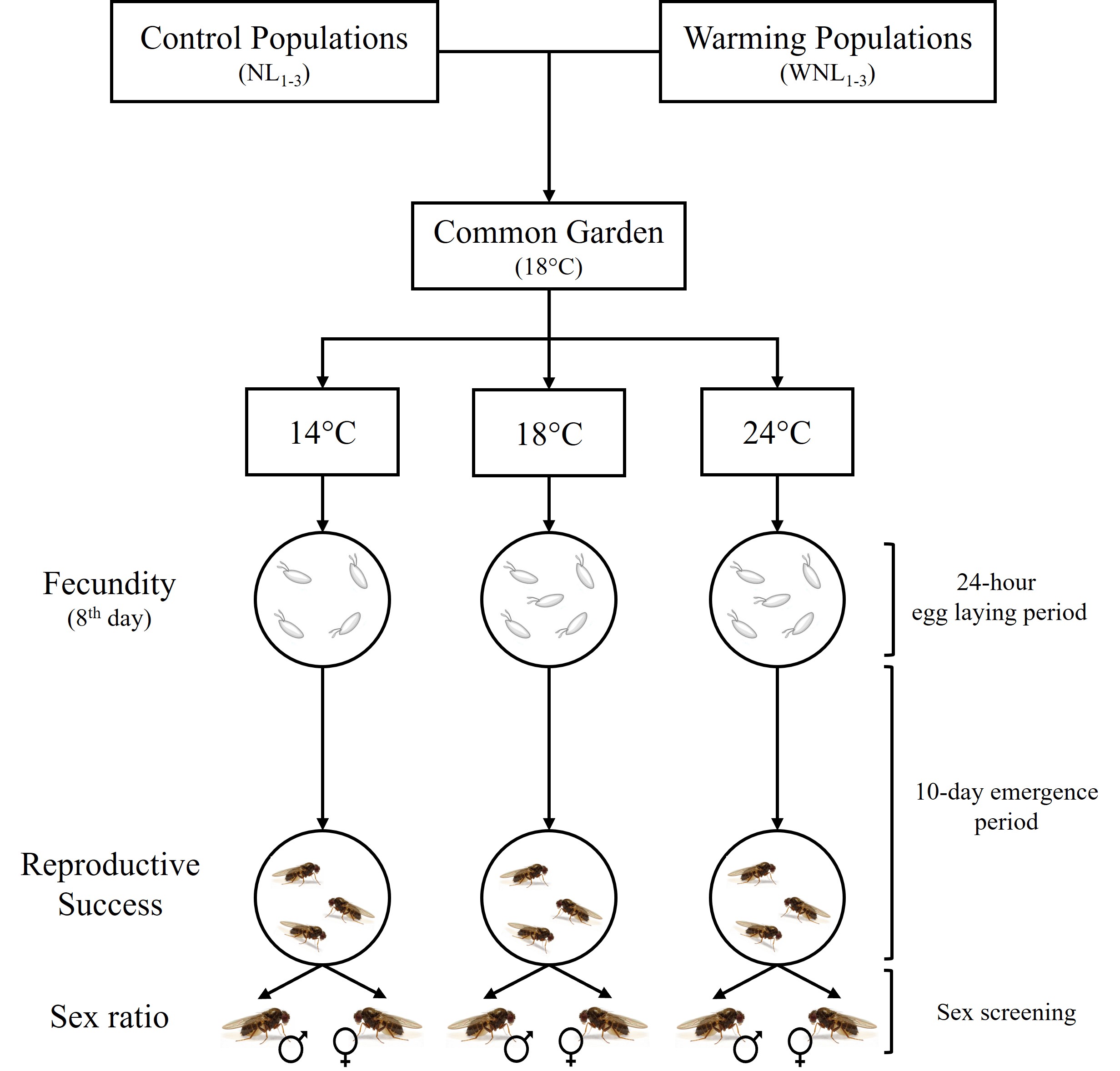

Supplement: Supplementary file 3 — Additional file 3: Figure S1. Schematic representation of the protocol used to assess the sex ratio after exposure to different thermal treatments. Legend: After 31 generations of thermal evolution individuals from both Control and Warming regimes were subjected to a one full generation common garden (18 ºC). After the common garden both selection regimes were submitted to one of three thermal treatments (14 °C, 18 °C or 24 °C). The emerging adults, formed in pairs, were assayed for fecundity at those same temperatures and the reproductive success was measured after a 10-day emergence period. In this study, a total of 6103 individuals were screened to assess the populations’ sex ratio. [file 12862_2023_2172_MOESM3_ESM.jpg]
